# A nomogram prediction model based on clinicopathological combined radiological features for metachronous liver metastasis of colorectal cancer

**DOI:** 10.7150/jca.88778

**Published:** 2024-01-01

**Authors:** Zhihua Lu, Jinbing Sun, Mi Wang, Heng Jiang, Guangqiang Chen, Weiguo Zhang

**Affiliations:** 1 Department of Radiology, Dushu Lake Hospital Affiliated to Soochow University, Medical Center of Soochow University, Suzhou Dushu Lake Hospital, Suzhou, Jiangsu, 215123, China.; 2 Department of General Surgery, Changshu No.1 People's Hospital, Affiliated Changshu Hospital of Soochow University, 1 Shuyuan Road, Changshu, Jiangsu 215500, China.; 3 Soochow University, Suzhou, Jiangsu, 215031, China.; 4 Department of Radiology, the Second Affiliated Hospital of Soochow University, Suzhou, Jiangsu, 215004, China.

**Keywords:** colorectal cancer, metachronous liver metastasis, clinicopathological and radiological features, nomogram, prediction model

## Abstract

**Objective:** To establish a nomogram prediction model (based on clinicopathological and radiological features) for the development of metachronous liver metastasis (MLM) in patients with colorectal cancer (CRC).

**Methods:** This retrospective study included patients with CRC who underwent surgery at Changshu No.1 People's Hospital and the Second Affiliated Hospital of Soochow University between January 2016 and December 2018. The clinical, pathological, and radiological features of each patient were investigated. Risk factors for MLM were identified by univariable and multivariable analyses. The predictive nomogram for MLM development was constructed. The predictive performance of the nomogram was estimated by the receiver operating characteristics curve, calibration curve, and decision curve analysis.

**Results:** This study included 161 patients with CRC [median age: 66 (range, 33-87) years]. Fifty-nine developed MLM after a median of 12 (range, 2-52) months after surgery. The multivariable logistic regression analysis showed that age >66 years (OR=3.471, 95% CI: 1.272-9.473, *P*=0.015), N2 stage (OR=6.534, 95% CI: 1.456-29.317, *P*=0.014), positive vascular invasion (OR=2.995, 95% CI: 1.132-7.926, *P*=0.027), positive tumor deposit (OR=4.451, 95% CI: 1.153-17.179, *P*=0.030), and linear (OR=6.774, 95% CI: 1.306-35.135, *P*=0.023) and nodal pericolic fat infiltration patterns (OR=8.762, 95% CI: 1.521-50.457, *P*=0.015) were independently associated with MLM. These five factors were used to create a nomogram. The area under the receiver operating characteristics curve of the nomogram was 0.866 (95% CI: 0.803-0.914), indicating favorable prediction performance. The calibration curve of the nomogram showed a satisfactory agreement between the predicted and actual probabilities.

**Conclusions:** A nomogram prediction model based on five clinicopathological and radiological features might have favorable prediction performance for MLM in patients who underwent surgery for CRC. Hence, the present study proposes a nomogram that can easily be used to predict MLM after CRC surgery based on readily available features.

## Introduction

Colorectal cancer (CRC) is a malignant neoplasm of the colon or rectum and the third most common cancer worldwide, with an estimated 1,880,725 new cases worldwide in 2020 and 915,880 related deaths 1. The liver is the most common site of distant metastasis of CRC 2-4. Liver metastasis (LM) is difficult to manage and the main cause of death among patients with CRC. The 5-year survival rate of patients with unresectable LM was less than 5% 2,5. About 15%-30% of patients with CRC already have LM at CRC diagnosis (i.e., synchronous), and 20%-50% will develop metachronous LM (MLM) after radical resection of the primary tumor 3. The early identification of patients with CRC at a high risk of MLM is essential for targeted screening and individualized treatment. Therefore, it is meaningful to identify MLM high-risk features.

Previous studies reported that some clinical and pathological risk factors are independent factors for LM of CRC, including age, preoperative serum carcinoembryonic antigen (CEA) levels, T and N stages, vascular invasion, histological grade, and KRAS mutations 6-11, but there are no consensuses. In addition, effective imaging characteristics of the primary CRC for predicting MLM are lacking. Kim et al. 12 analyzed the computed tomography (CT) features of primary CRC, including the morphologic and enhancement characteristics; seven CT features were associated with poorly differentiated (PD) over well- or moderately differentiated (WD or MD) CRC. Eurboonyanun et al. 13 reported that BRAF-mutant CRC has specific imaging characteristics. Hence, the CT features of the primary CRC could helpfully provide effective baseline imaging characteristics for prognosis. In addition, the imaging characteristics of the primary CRC are often less affected and more stable 14. Therefore, it can be hypothesized that some CT features of primary CRC tumors may be helpful in predicting MLM in patients with CRC.

The present study aimed to establish a nomogram model based on clinicopathological and radiological features for predicting MLM from CRC.

## Methods

### Study design and patients

This retrospective study included patients with CRC treated by surgical resection at Changshu No.1 People's Hospital and the Second Affiliated Hospital of Soochow University between January 2016 and December 2018.

The inclusion criteria were 1) underwent CRC resection and 2) histopathologically confirmed colorectal adenocarcinoma. The exclusion criteria were 1) mucinous adenocarcinoma, 2) synchronous liver metastasis, 3) the first metastatic sites did not include the liver, 4) incomplete clinical and radiological data, 5) neoadjuvant therapy or 6) follow-up of less than 3 years.

This study was approved by the Ethics Committee of Changshu No.1 People's Hospital (approval #(2020) LUN No. 012). The first author, Zhihua Lu, originally worked in the Changshu No.1 People's Hospital and was transferred to Dushu Lake Hospital Affiliated to Soochow University, after completing the project. The study was conducted in accordance with the Declaration of Helsinki. The requirement for individual informed consent was waived by the board.

### Data collection and definitions

The pathological diagnosis of the included cases in this study was consistent with the AJCC Version 8 diagnostic criteria 15. Although different definitions of MLM have been suggested 16-19, the present study used diagnosis/surgery as the cut-off point between the 'synchronous' and 'metachronous' groups 19.

The clinical information and pathological data of each patient were collected from the electronic patient charts. The clinical information included the age, sex, preoperative serum CEA (normal CEA: 0-10 ng/ml), preoperative carbohydrate antigen 19-9 (CA19-9) (normal CA19-9: 0-37 U/ml), primary tumor site, and CT scan data of the chest, abdomen, and pelvis. The pathological information included T and N stages, histologic tumor grade, vascular invasion, perineural invasion, and tumor deposits.

The following features were dichotomized into two categories: sex (male vs. female), age (median of the study population; ≤ 66 vs. > 66 years), preoperative CEA levels (upper limit of the normal range; ≤ 10 vs. >10 ng/ml), preoperative CA19-9 levels (upper limit of the normal range; ≤ 37 vs. > 37 µ/ml), vascular invasion (no vs. yes), perineural invasion (no vs. yes), tumor deposits (no vs. yes), maximal wall thickness (median of the study population; ≤ 15 vs. > 15 mm), tumor shape (according to the literature 12,13; thicken vs. polypoid or bulky), and colonic obstruction (no vs. yes). The other features were classified as multiple categories: T stage (T_1-2_, T_3_, and T_4_), N stage (N_0_, N_1_, and N_2_), differentiation grade (well, moderately, and poorly), enhancement pattern (homogeneous, heterogeneous ≤50%, and heterogeneous >50%), enhancement degree (higher attenuation than the liver, attenuation between the liver and muscle, and lower attenuation than the muscle), pericolic fat infiltration pattern (normal, hazy, linear, and nodular), maximal size of regional LN (< 5, 5-10, and > 10 mm).

### Postoperative follow-up

The postoperative clinical follow-up was performed according to the Chinese guidelines 20. The follow-up examinations were performed for 3 years, including physical examination, abdominal ultrasound, serum CEA, and CA19-9. For patients with stage II or III CRC, a contrast-enhanced CT scan of the chest, abdomen, and pelvis was performed once a year in the first 3-5 years and once every 1-2 years in the following years. For patients who were highly suspected of liver metastases on CT images but could not be diagnosed definitely, liver magnetic resonance imaging (MRI) was performed.

### Computed tomography features

The analysis of the CT features was based on the methods by Kim et al. 12 and Eurboonyanun et al. 13. The analysis included 1) maximal wall thickness, 2) shape of the tumor, 3) enhancement pattern of the tumor, 4) enhancement degree of the tumor, 5) colonic obstruction, 6) pericolic fat infiltration pattern, and 7) size of the regional lymph nodes (LNs).

The maximal wall thickness was measured on images perpendicular to the long axis of the tumor. The tumor's shape was classified as intraluminal polypoid mass or bulky and wall thickening (**Figure S1**).

The enhancement pattern of the tumor was evaluated in the portal venous (PV) phase and classified as homogeneous vs. heterogeneous. The heterogeneous pattern was demonstrated as lower attenuation to the tumor due to cystic change, necrosis, and mucinous components in the tumor. According to the ratio of low attenuation area to the tumor, it was further divided into heterogeneous ≤ 50% vs. heterogeneous > 50% (**Figure S2**).

The enhancement degree of the tumor was evaluated in the PV phase and classified as higher attenuation than the liver, attenuation between the liver and muscle, and lower attenuation than the muscle; the region of interest (ROI) was drawn by selecting the largest layer of the solid part of the mass and sketching as much solid part as possible.

The average CT value of the tumor was measured on the largest tumor image and compared with the hepatic parenchyma and muscle; the ROI was drawn by selecting the largest dimension of the solid portion of the mass and sketching the maximum solid portion measurement with obvious enhancement. Colonic obstruction was classified as yes or no according to the CT features.

Pericolic fat infiltration pattern was classified as normal, hazy, linear, and nodular (**Figure S3**). If the outer contour of the tumor-bearing colorectal segment was smooth and the mesentery adjacent to the tumor showed the same appearance as the adjacent intra-abdominal fat, then it was considered normal. If the outer contour of the tumor-bearing colorectal segment was smooth and the mesentery adjacent to the tumor showed ill-defined, slightly increased density, then it was considered hazy. If the outer layer of the tumor-bearing colorectal segment was coarse and the mesentery adjacent to the tumor showed a well-defined, linear configuration, then it was considered linear. If the outer contour of the tumor-bearing colorectal segment showed a well-defined nodular configuration and invaded into peritumoral mesentery, then it was considered nodular.

The size of the regional LNs was classified as no visible LNs, < 5 mm, 5-10 mm, and > 10 mm according to the short-axis diameter. Regional LNs were defined as LNs located along the course of the major vessels supplying the tumor-bearing colorectum, along the vascular arcades of the marginal artery, and the mesocolic border of the colon 21.

### Statistical analysis

Statistical analysis was performed using SPSS 22.0 (IBM Corp., Armonk, NY, USA). The nomogram was plotted using the “rms” package in R version 3.4.1, and all ROC curves were drawn using MedCalc 18.0. All data were expressed as n (%).

The categorical variables were analyzed using the chi-square test. The patient characteristic variables with statistical significance (P < 0.05) between the two groups were included in the multivariable logistic regression analysis to identify the independent risk factors for MLM. A predictive nomogram for MLM development was constructed based on the independent risk factors screened by multivariable logistic analysis. The predictive efficiency of the nomogram model was evaluated using the receiver operating characteristics (ROC) method, and the area under the curve (AUC) was calculated. Finally, the calibration curve of the nomogram was performed. In order to evaluate the nomogram in clinical application value, decision curve analysis (DCA) was used to calculate net benefit under the probability of each risk threshold.

## Results

**Figure 1:**
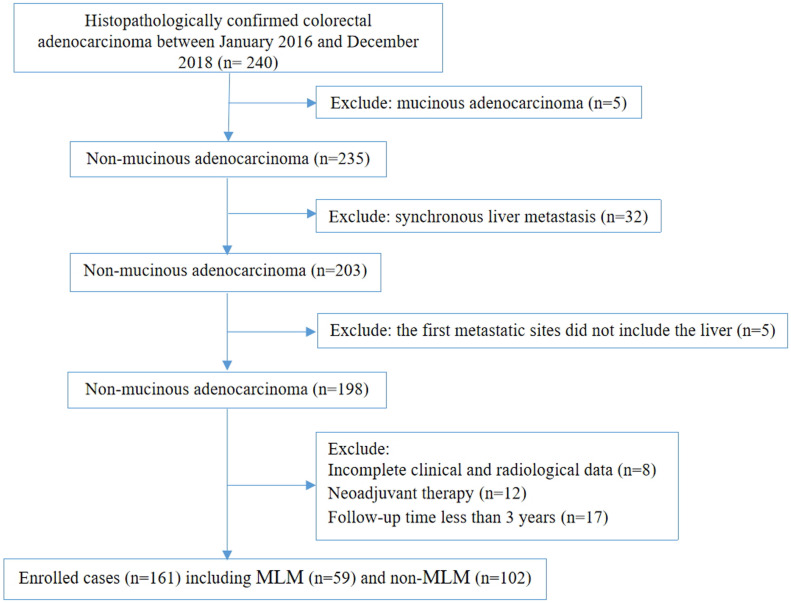
Flow chat of recruitment, inclusion, and exclusion.

This study included 161 patients with CRC [median age: 66 (range, 33-87) years] (**Figure 1**); 59 developed MLM in a median of 12 (range, 2-52) months after surgery. Among 17 characteristics, 10 were significantly different between the MLM and non-MLM groups, including age (*P* = 0.036), T stage (*P* = 0.037), N stage (P < 0.001), vascular invasion (*P* < 0.001), maximal wall thickness (*P* = 0.015), enhancement pattern (*P* = 0.005), tumor deposit (*P* < 0.001), colonic obstruction (*P* = 0.023), pericolic fat infiltration pattern (*P* < 0.001), and maximal size of regional LNs (*P* = 0.014) (**Table 1**). Sex (*P* = 0.714), preoperative CEA levels (*P*=0.104), preoperative CA199 levels (*P* = 0.563), perineural invasion (*P* = 0.179), differentiation grade (*P* = 0.070), tumor shape (*P* = 0.735), and enhancement degree (*P* = 0.526) were not associated with MLM.

The multivariable logistic regression analysis showed that age >66 years (OR = 3.471, 95% CI: 1.272-9.473, *P* = 0.015), N2 stage (OR = 6.534, 95% CI: 1.456-29.317, *P* = 0.014), positive vascular invasion (OR = 2.995, 95% CI: 1.132-7.926, *P* = 0.027), positive tumor deposit (OR = 4.451, 95% CI: 1.153-17.179, *P* = 0.030), and linear (OR = 6.774, 95% CI: 1.306-35.135, *P* = 0.023) and nodal (OR = 8.762, 95% CI: 1.521-50.457, *P* = 0.015) pericolic fat infiltration patterns were independently associated with MLM (**Table 2**). There was no collinearity among the five variables (**Figure S4**).

**Figure 2:**
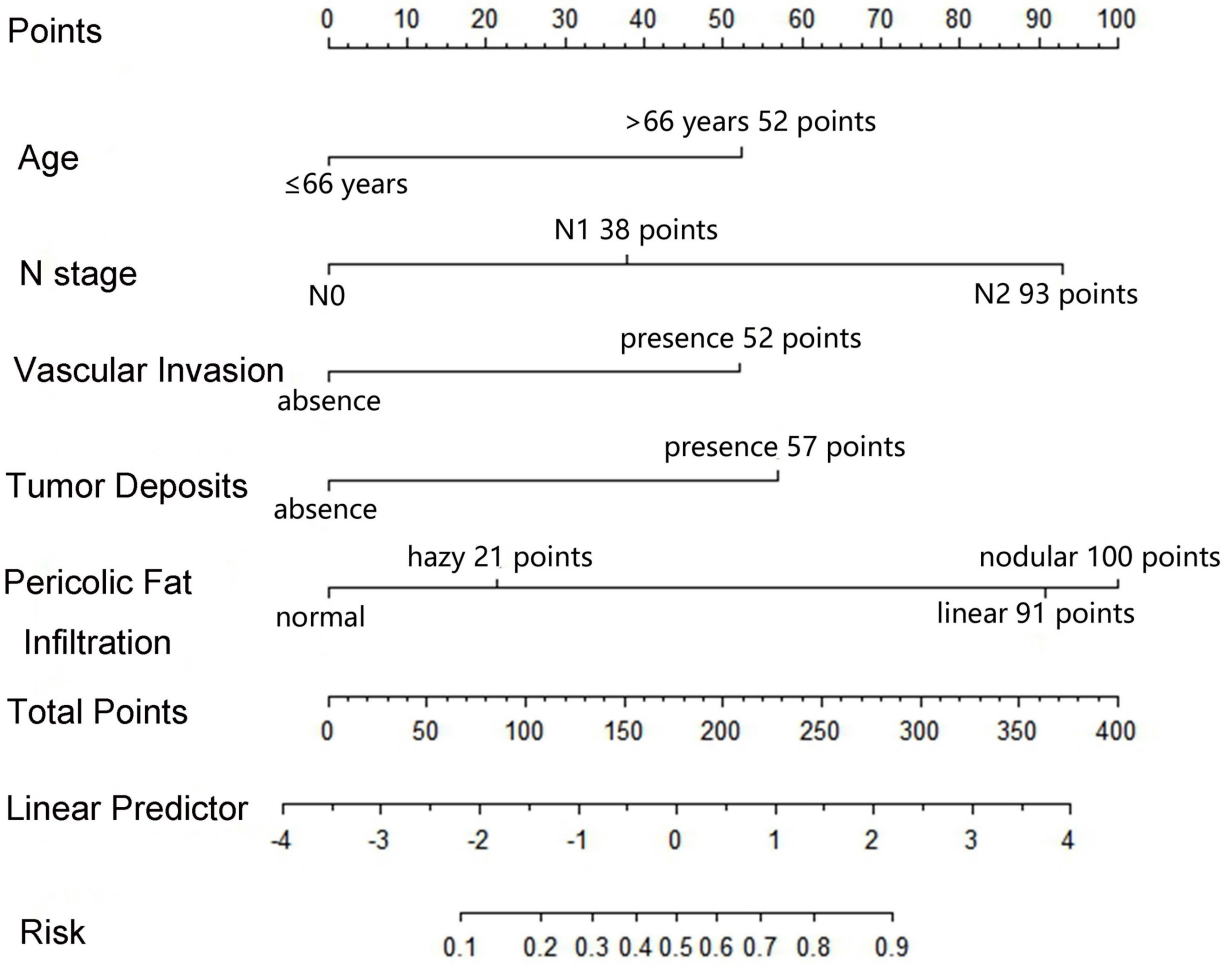
Nomogram for predicting the development of MLM. For each variable, a vertical line is drawn from the value to the upper “Points” axis to determine the score of each variable. Then, the scores are added together, and a vertical line is drawn from the “Total Points” axis to the “Risk” axis to determine the risk of MLM.

**Figure 3:**
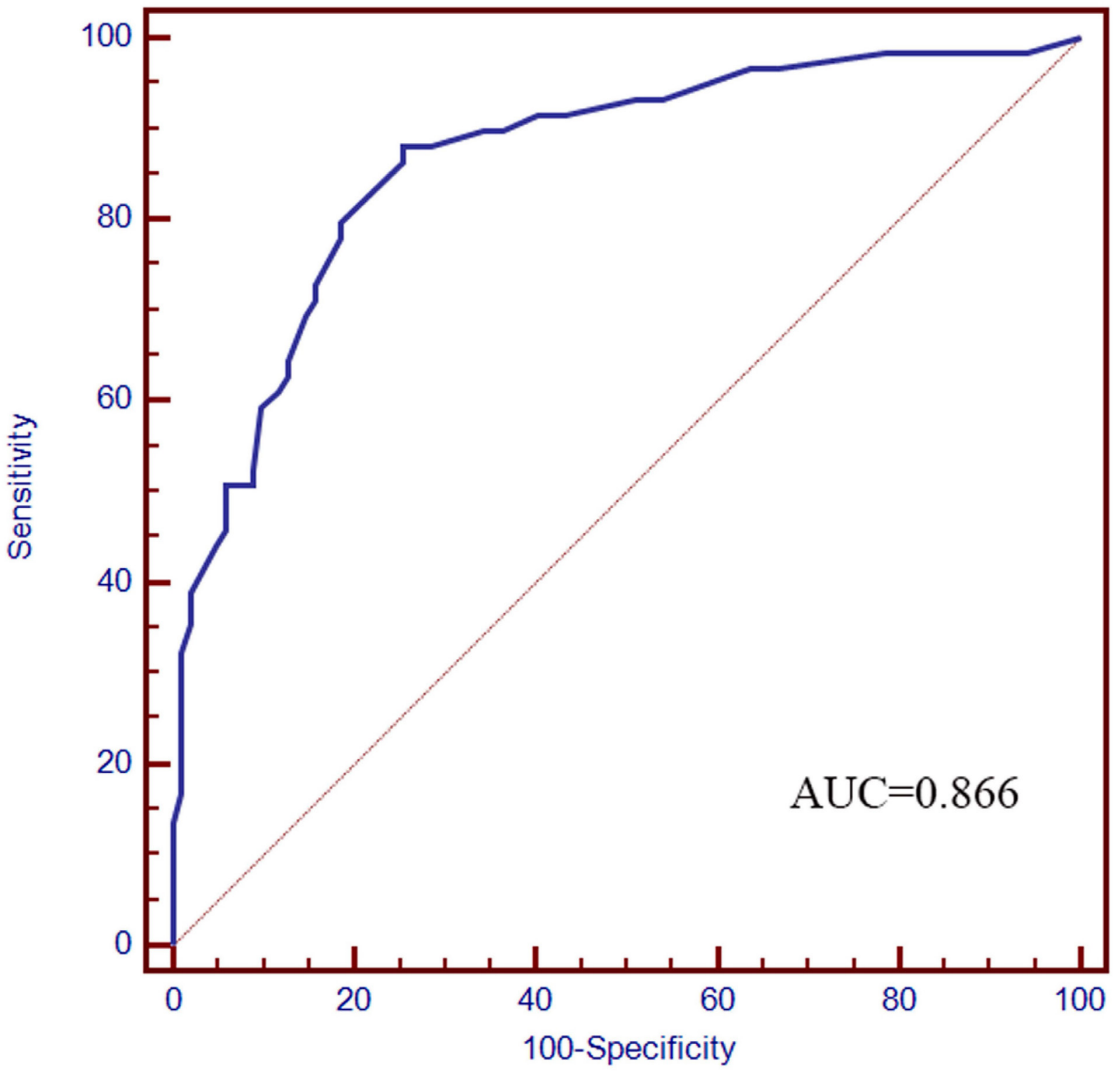
ROC curve. The AUC was 0.866 (95% CI: 0.803-0.914).

**Figure 4:**
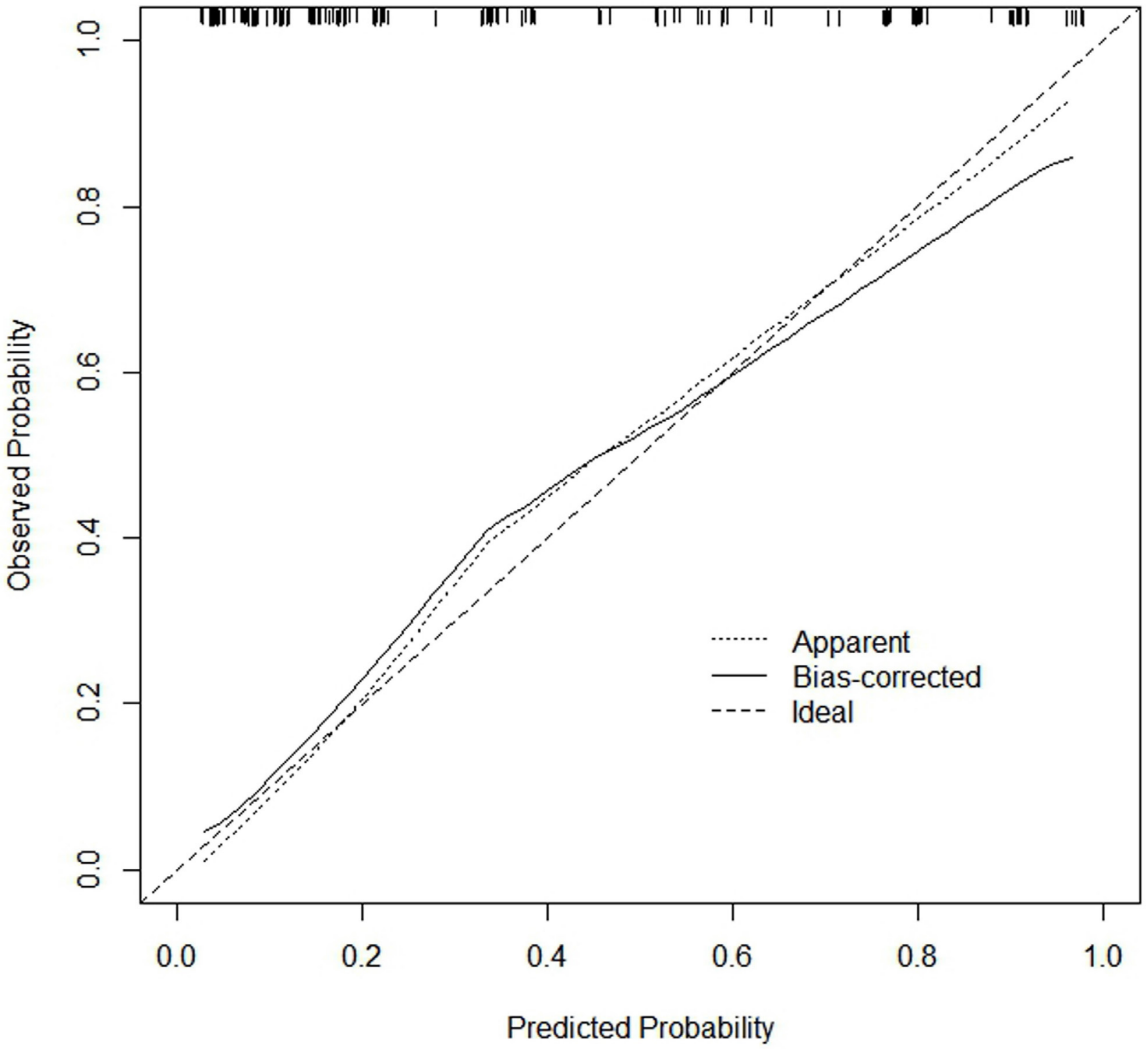
Calibration curve of the nomogram. The x-axis represents the predicted MLM probability. The y-axis represents the actual diagnosed MLM probability.

**Figure 5:**
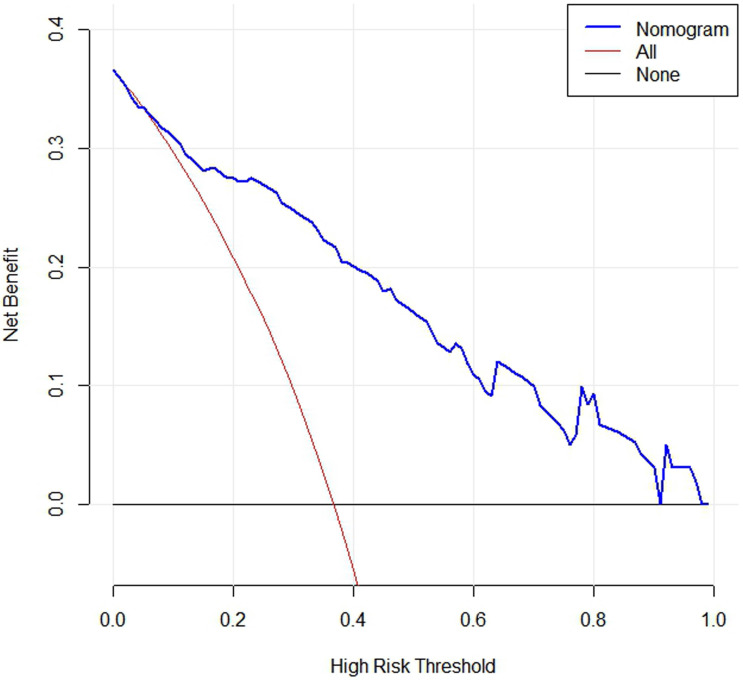
DCA curve of the nomogram. When the risk threshold probability is >1% and <91%, using this nomogram to identify MLM could achieve a net clinical beneft.

Based on the five factors independently associated with MLM, a nomogram was developed for predicting MLM of CRC (**Figure 2**). The AUC of the ROC curve was 0.866 (95% CI: 0.803-0.914, *P* < 0.001) (**Figure 3**), meaning that the nomogram model had good predictive efficiency. The nomogram had 88.1% sensitivity, 74.5% specificity, 79.5% accuracy, 73.2% positive predictive value, and 82.9% negative predictive value. In addition, the calibration curve of the nomogram showed a satisfactory agreement between predicted and actual probability (**Figure 4**). **Figure 5** shows the decision curve analysis.

Of the 161 patients in this study, 118 had available postoperative treatment records at our hospital (43 did not receive adjuvant therapy or received it in other hospitals). Among the 118 patients, the final TNM staging was II, III, and IV in 48, 66, and four. Regarding adjuvant therapy, 83 received FOLFOX, and 35 received CapeOx for 3-6 months. Five patients with rectal cancer received postoperative adjuvant chemotherapy and pelvic radiation therapy.

## Discussion

The results showed that a nomogram prediction model based on age >66 years, N2 stage, positive vascular invasion, positive tumor deposit, and linear and nodal pericolic fat infiltration patterns might have favorable prediction performance for the progression of CRC to MLM. These findings may help clinicians identify patients with a high risk of MLM development after CRC resection and adjust the follow-up accordingly. The present study proposes a nomogram that can easily be used to predict MLM after CRC surgery based on readily available features.

Previous studies focused on the clinical and pathological factors that could predict MLM in CRC patients 7,8,11, but few studies included the CT imaging characteristics of the primary CRC to predict the development of MLM. Xiao et al. 22 constructed a model based on deep learning analysis of pathological images. None of these models included imaging features, while the present study did. Recent studies used radiomics to construct a model predicting MLM after CRC resection 23-26, but radiomics relies heavily on the software and local imaging parameters used for scanning. The naked eye cannot observe most radiomics features, and the external validity is generally limited 27. It is why it was decided to examine hard CT features in the present study.

Age and CEA have been confirmed to be important clinical risk factors for developing MLM of CRC 7,8,11. The present study demonstrated that age was independently associated with the development of MLM. Previous studies reported the association between preoperative serum CEA levels and LM, prognosis, and recurrence of patients with CRC 7,8,11. Generally, increased serum CEA levels before surgery have been related to MLM of CRC 28,29. Nevertheless, preoperative serum CEA was not associated with MLM in the univariable logistic regression analysis, which might be because serum CEA levels are influenced by several factors 28,29, including tumor size, tumor CEA contents, CEA production rates, tumor location, and the rate of CEA elimination 29-31, and their results contradict the suggestion that CEA levels increase with more advancing stages of CRC.

Previous studies reported that pathological factors such as higher T stage, positive N stage, and positive vascular invasion were associated with MLM of CRC 7-11. A higher T stage means that the tumor cells infiltrate the intestinal wall deeper, resulting in a higher probability of infiltration of blood vessels and lymphatic vessels and a higher risk of distant metastasis. In the multivariable logistic regression analysis, the N2 stage and positive vascular invasion were independently associated with MLM. In contrast, the T stage was associated with the univariable analysis but not the multivariable one. The limited sample size could explain these results. In this study, 23% of the patients with the T1-2 stage (n = 7), 47% of the patients with the T3 stage (n = 33), and 32% of the patients with the T4 stage (n = 19) developed MLM.

Tumor deposits are defined as discrete tumor nodules without histologic evidence of a residual LN identified in the pericolic or perirectal tissue away from the leading edge of the tumor 32. In the latest 7^th^ and 8^th^ editions of the American Joint Committee on Cancer (AJCC) TNM staging system, if there is a positive tumor deposit but no concurrent LN metastasis, the N stage should be categorized as the N1c stage 32. Positive tumor deposits are associated with recurrence, metastasis, and poorer survival outcomes in CRC patients 33-35. In the present study, positive tumor deposit was independently associated with the development of MLM by multivariable logistic regression analysis. In line with this result, Wu et al. 33 retrospectively analyzed two large independent cohorts of patients with CRC and showed that tumor deposits were an independent predictor of liver metastasis (OR: 4.662, CI:2.743-7.923). For CRC patients whose postoperative pathological results suggest the presence of positive tumor deposits, more rigorous follow-up might be needed.

Current studies on predicting MLM of CRC based on imaging of liver parenchyma or primary tumor are rare. Beckers et al. 24,25 analyzed the CT images of the liver parenchyma of patients with CRC based on texture analysis and got limited results. Indeed, uniformity had the potential to predict LM during the first postoperative 6 months but not beyond 6 months 25. In addition, there were no additional effects found for texture assessment on a segmental level 24. Still, several factors may influence imaging measurement of the liver parenchyma 25. On the other hand, the imaging characteristics of the primary CRC tumor are often less affected and more stable than liver parenchyma 14. After multivariable logistic regression analysis, the pericolic fat infiltration pattern was considered the most important predictor in the nomogram model. The linear and nodular patterns also indicated a higher risk of MLM. The explanation might be that the linear and nodular patterns are associated with deeper infiltration, resulting in more vascular invasion and LN metastasis. Previous studies reported that pericolic fat infiltration was correlated with the pathological variables of CRC 12,36,37. A study by Kim et al. 12 reported that PD colorectal adenocarcinoma demonstrated significantly more nodular pericolic fat infiltration than WD or MD. Sa et al. 36 analyzed the correlation between the T stage of CRC and nine CT imaging characteristics. Six CT variables, including pericolic fat infiltration, were positively correlated with the T stage. Zeina et al. 37 quantitatively analyzed the pericolic fat, including the maximal distance between tumor margins and normally appearing mesenteric fat and mean CT values of pericolic fat adjacent to the tumor. They found that the overall sensitivity, specificity, and accuracy of pericolic fat infiltration in detecting patients with ≥T3 stage were 95%, 20%, and 81.9%. Still, Ng et al. 38 reported that abnormal pericolic fat features were not a precise indicator of the extramuscular extension of the tumor. Therefore, the pericolic fat infiltration pattern of the primary CRC tumor, especially linear and nodular patterns, might help predict the development of MLM.

There were some limitations in this study. Firstly, the sample size was limited. Secondly, all patients were followed up for at least 3 years, but the follow-up was relatively short (<5 years). Therefore, it is possible that a longer follow-up would yield more cases of MLM. Furthermore, patients with advanced CRC generally receive chemotherapy after surgery, and the development of MLM might be affected by chemotherapy. It will have to be considered in future larger studies. Thirdly, although the nomogram showed good predictive efficiency, this study only used internal data. External validity remains to be evaluated. Fourthly, there are some molecular tumor markers, such as KRAS, NRAS, and BRAF, that might have promising relevance between LM and their positive value 39,40, but it was impossible to collect these data in the present study because those biomarkers were not performed in all cases during the study period. Fifthly, the CT features analyzed in this study were based on the features by Kim et al. 12 and Eurboonyanun et al. 13, including basic morphological and enhancement characteristics, but they might not be comprehensive enough. Other detailed CT features of primary CRC should be examined in the future.

## Conclusion

This study established a nomogram model for predicting the risk of MLM development in patients with CRC based on clinical and pathological features (age, N stage, vascular invasion, and tumor deposits) and a radiological feature (pericolic fat infiltration pattern) with good calibration and good predictive efficiency. This model might help clinicians identify patients with a high risk of MLM development after CRC resection and adjust the follow-up accordingly.

## Supplementary Material

Supplementary figures and tables.

## Figures and Tables

**Table 1:** Basic characteristics.

Variables	Without MLM (n=102)	With MLM (n=59)	*χ^2^*	*P*
Sex, n (%)			0.135	0.714
Male	54 (52.9)	33 (55.9)		
Female	48 (47.1)	26 (44.1)		
Age, n (%)			4.387	0.036
≤66 years	64 (62.7)	27 (45.8)		
>66 years	38 (37.3)	32 (54.2)		
Preoperative CEA levels (n=153) ^a^, n (%)			2.649	0.104
≤10 ng/ml	71 (69.6)	35 (59.3)		
>10 ng/ml	25 (24.5)	22 (37.3)		
Preoperative CA199 levels (n=150) ^b^, n (%)			0.335	0.563
≤37 U/ml	88 (86.3)	51 (86.4)		
>37 U/ml	6 (5.9)	5 (8.5)		
T stage, n (%)			6.604	0.037
T_1-2_	24 (23.5)	7 (11.9)		
T_3_	37 (36.3)	33 (55.9)		
T_4_	41 (40.2)	19 (32.2)		
N stage, n (%)			33.184	<0.001
N_0_	71 (69.6)	15 (25.4)		
N_1_	26 (25.5)	28 (47.5)		
N_2_	5 (4.9)	16 (27.1)		
Vascular invasion, n (%)			22.469	<0.001
No	78 (76.5)	23 (39.0)		
Yes	24 (23.5)	36 (61.0)		
Perineural invasion, n (%)			1.807	0.179
No	79 (77.5)	40 (67.8)		
Yes	23 (22.5)	19 (32.2)		
Differentiation grade, n (%)			5.323	0.070
Well-differentiated	8 (7.8)	1 (1.7)		
Moderately differentiated	77 (75.5)	41 (69.5)		
Poorly differentiated	17 (16.7)	17 (28.8)		
Tumor deposits, n (%)			27.528	<0.001
Yes	12 (11.8)	29 (49.2)		
No	90 (88.2)	30 (50.8)		
Maximal wall thickness, n (%)			5.891	0.015
≤15 mm	60 (58.8)	23 (39.0)		
>15 mm	42 (41.2)	36 (61.0)		
Tumor shape, n (%)			0.114	0.735
Thicken	88 (86.3)	52 (88.1)		
Polypoid or bulky	14 (13.7)	7 (11.9)		
Enhancement pattern, n (%)			10.606	0.005
Homogeneous	60 (58.8)	19 (32.2)		
Heterogeneous ≤50%	28 (27.5)	27 (48.5)		
Heterogeneous >50%	14 (13.7)	13 (22.0)		
Enhancement degree, n (%) ^c^			0.401	0.526
Attenuation between the liver and muscle	90 (88.2)	50 (84.7)		
Lower attenuation compared with the muscle	12 (11.8)	9 (15.3)		
Colonic obstruction, n (%)			5.197	0.023
Yes	17 (16.7)	19 (32.2)		
No	85 (83.3)	40 (67.8)		
Pericolic fat infiltration, n (%)			31.580	<0.001
Normal	28 (27.5)	3 (5.1)		
Hazy	37 (36.3)	8 (13.6)		
Linear	23 (22.5)	26 (44.1)		
Nodular	14 (13.7)	22 (37.3)		
Maximal size of regional lymph node, n (%)			8.564	0.014
<5 mm	46 (45.1)	19 (32.2)		
5-10 mm	47 (46.1)	25 (42.4)		
>10 mm	9 (8.8)	15 (25.4)		

^a^ 8 cases missing data; ^b^ 11 cases missing data; ^C^ no cases of higher attenuation than the liver. MLM: metachronous liver metastasis; CEA: carcinoembryonic antigen; CA: carbohydrate antigen.

**Table 2:** Univariable and multivariable logistic regression analysis of the risk features for postoperative liver metastasis

Variables	Univariable logistic regression analysis			Multivariable logistic regression analysis		
	OR	95%CI	P	OR	95%CI	P
Sex						
Females	Ref.	-	-			
Males	1.128	0.592, 2.149	0.714			
Age						
≤ 66	Ref.	-	-	Ref.	-	-
>66	1.996	1.041, 3.826	0.036	3.471	1.272-9.473	0.015
T stage						
T_1-2_	Ref.	-	-	Ref.	-	-
T_3_	3.058	1.166, 8.017	0.023	1.374	0.355-5.320	0.645
T_4_	1.589	0.583, 4.329	0.365	0.531	0.125-2.253	0.391
N stage						
N_0_	Ref.	-	-	Ref.	-	-
N_1_	5.097	2.357, 11.025	< 0.001	1.726	0.479-6.217	0.404
N_2_	15.147	4.804, 47.756	< 0.001	6.534	1.456-29.317	0.014
Vascular Invasion						
No	Ref.	-	-	Ref.	-	-
Yes	5.087	2.539, 10.193	< 0.001	2.995	1.132-7.926	0.027
Perineural Invasion						
No	Ref.	-	-			
Yes	1.632	0.797, 3.341	0.179			
Differentiation grade						
Well-differentiated	Ref.	-	-			
Moderately differentiated	4.260	0.515, 35.245	0.179			
Poorly differentiated	8.000	0.900, 71.115	0.062			
Tumor shape						
Polypoid or bulky	Ref.	-	-			
Thicken	0.846	0.321, 2.232	0.735			
Maximal Wall Thickness						
≤15 mm	Ref.	-	-	Ref.	-	-
>15 mm	2.236	1.161, 4.305	0.015	0.774	0.250-2.401	0.658
Enhancement Pattern of Tumor						
Homogeneous	Ref.	-	-	Ref.	-	-
Heterogeneous<50%	3.045	1.455, 6.374	0.003	4.017	1.102-13.207	0.056
Heterogeneous>50%	2.932	1.175, 7.317	0.021	3.318	0.856-12.864	0.083
Enhancement degree						
Lower attenuation compared with the muscle	Ref.	-	-			
Attenuation between the liver and muscle	1.350	0.532, 3.424	0.526			
Tumor Deposits						
No	Ref.	-	-	Ref.	-	-
Yes	7.250	3.292, 15.967	<0.001	4.451	1.153-17.179	0.030
Colonic Obstruction						
No	Ref.	-	-	Ref.	-	-
Yes	2.375	1.117, 5.051	0.023	2.127	0.703-6.438	0.182
Pericolic Fat Infiltration						
Normal	Ref.	-	-	Ref.	-	-
Hazy	2.018	0.490, 8.306	0.331	1.177	0.221-6.269	0.848
Linear	10.551	2.829, 39.347	< 0.001	6.774	1.306-35.135	0.023
Nodular	14.667	3.741, 57.503	< 0.001	8.762	1.521-50.457	0.015
Maximal Size of Regional Lymph Node						
< 5 mm	Ref.	-	-	Ref.	-	-
5-10mm	1.288	0.626, 2.651	0.492	0.493	0.153-1.590	0.237
> 10mm	4.035	1.508, 10.796	0.005	0.514	0.110-2.414	0.399
Carcinoembryonic antigen (CEA)						
≤ 10 ng/ml	Ref.	-	-			
> 10 ng/ml	1.785	0.885, 3.600	0.104			
Carbohydrate antigen 19-9 (CA19-9)						
≤ 37 U/ml	Ref.	-	-			
> 37 U/ml	1.438	0.418, 4.949	0.563			
