# Supplementary material for: A nomogram prediction model based on clinicopathological combined radiological features for metachronous liver metastasis of colorectal cancer
Source: J Cancer. 2024 Jan 1;15(4):916–25. doi: 10.7150/jca.88778 (PMC10788726; doi:10.7150/jca.88778)
Supplement: Supplementary file 1 — Supplementary figures and tables. [file jcav15p0916s1.pdf]

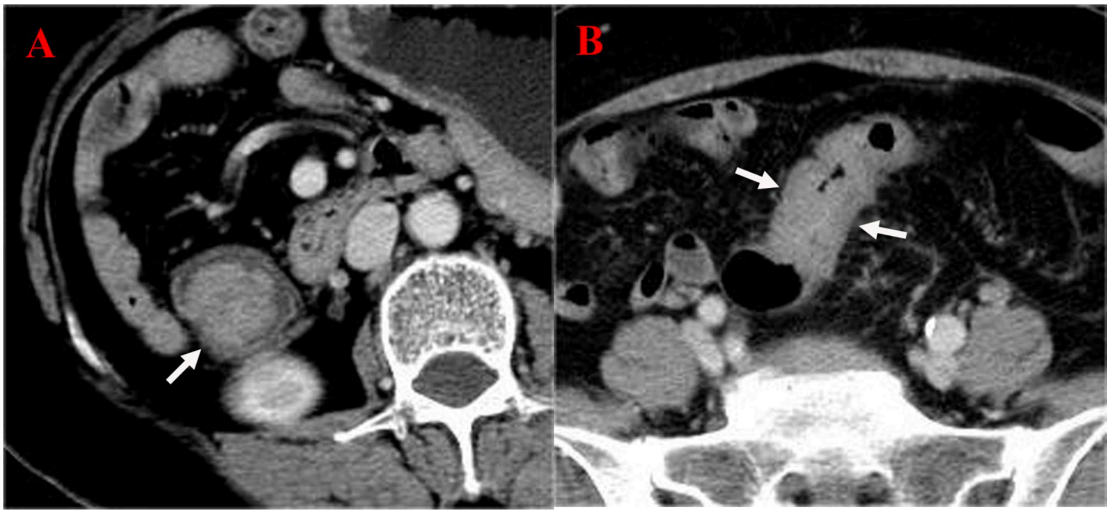

Supplementary Figure S1. Types of tumor shape. (A) Polypoid mass or bulky was defined as protruding mass into the colonic lumen with the tumor-bearing wall the same as the adjacent normal wall (arrow). (B) Wall thickening was defined as the diameter of the tumor-bearing segment being larger than the diameter of the normal distended colorectal segment (arrow).

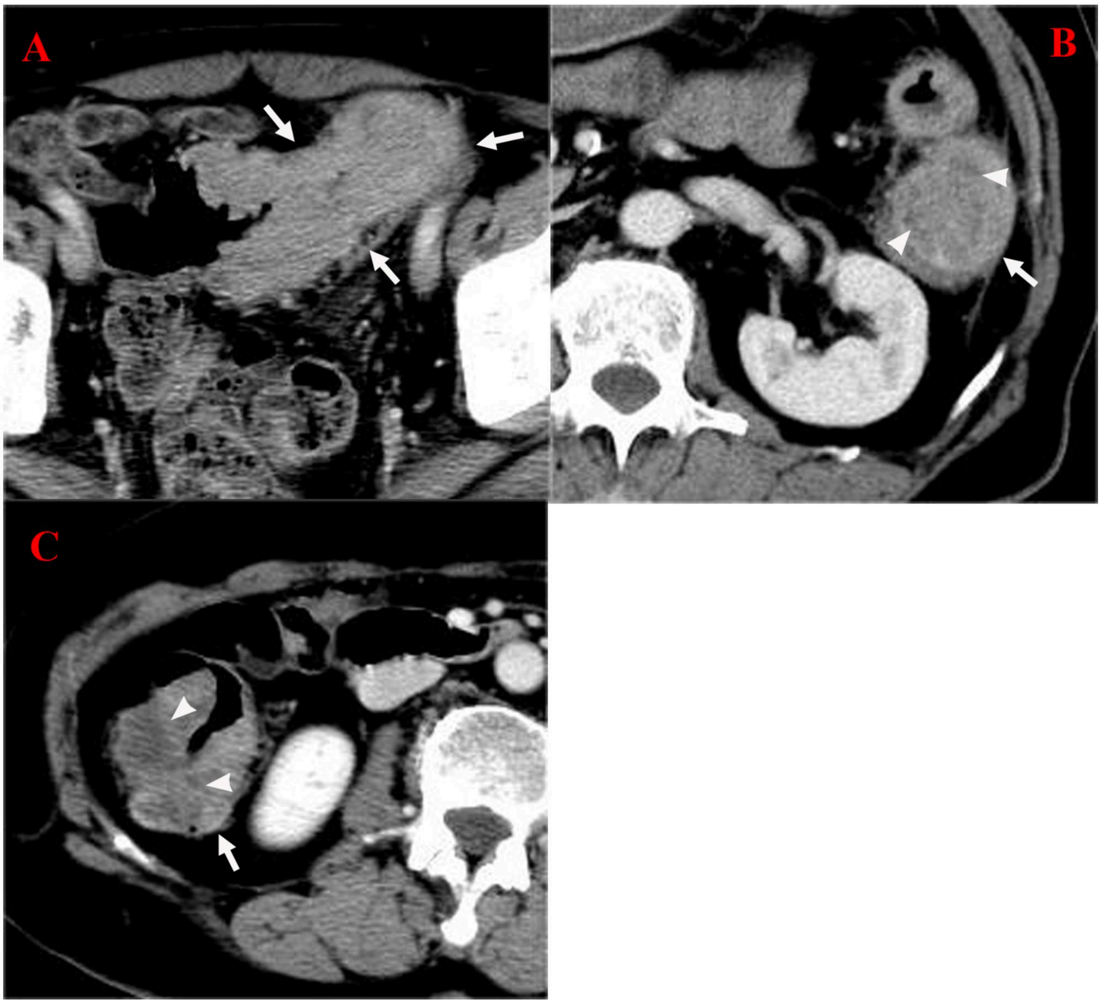

Supplementary Figure S2. Types of tumor enhancement pattern. (A) A homogeneous enhancement pattern was defined as the degree of uniform enhancement (arrow). (B) A heterogeneous ≤50% enhancement pattern was defined as the ratio of low attenuation (arrowhead) area to the tumor (arrow) ≤50%. (C) A heterogeneous >50% enhancement pattern was defined as the ratio of low attenuation (arrowhead) area to the tumor (arrow) >50%.

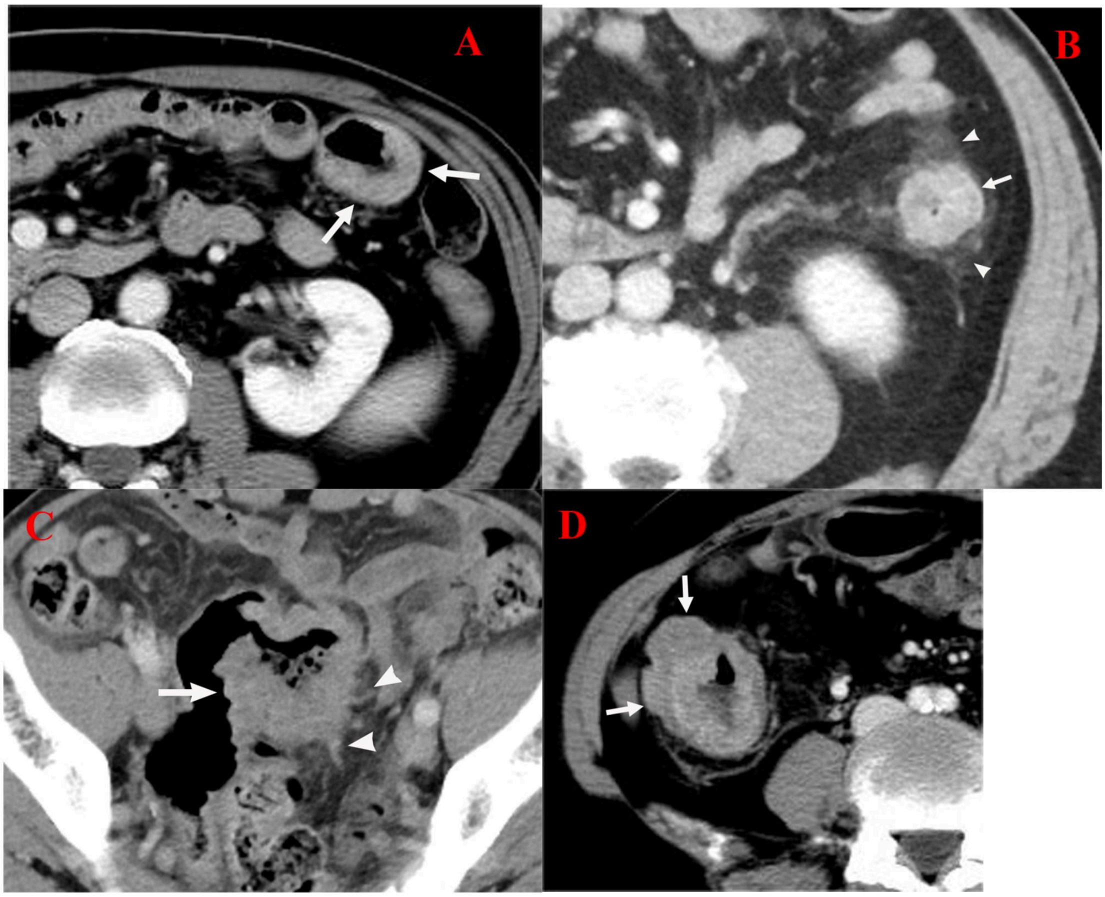

Supplementary Figure S3. Types of pericolic fat infiltration pattern. (A) Normal was defined as the outer contour of the tumor-bearing segment was smooth, and the adjacent mesentery showed the same appearance as the adjacent intra-abdominal fat (arrow). (B) Hazy was defined as the outer contour of the tumor-bearing segment (arrow) was smooth, and the adjacent mesentery showed ill-defined, slightly increased density (arrowhead). (C) Linear was defined as the outer layer of the tumor-bearing segment (arrow) was coarse, and the adjacent mesentery showed a well-defined, linear configuration (arrowhead). (D) Nodular was defined as the outer contour of the tumor-bearing segment that showed a well-defined nodular configuration and invaded into peritumoral mesentery (arrow).

Variables Entered/Removed[b]

Model	Variables Entered	Variables Removed	Method
1	PericolicFat Infiltration, age, Tumor Deposits, Vascular Invasion, Nstage	.	Enter

a. All requested variables entered.
b. Dependent Variable: LiverMetastasis

Model Summary

Model	R	R Square	Adjusted R Square	Std. Error of the Estimate
1	.618[a]	.382	.362	.386

a. Predictors: (Constant), PericolicFatInfiltration, age, TumorDeposits, VascularInvasion, Nstage

ANOVA[b]

Model		Sum of Squares	df	Mean Square	F	Sig.
1	Regression	14.272	5	2.854	19.147	.000[a]
	Residual	23.107	155	.149		
	Total	37.379	160			

a. Predictors: (Constant), PericolicFatInfiltration, age, TumorDeposits, VascularInvasion, Nstage
b. Dependent Variable: LiverMetastasis

Coefficients[a]

Model		Unstandardized Coefficients		Standardized Coefficients	t	Sig.	Collinearity Statistics	
		B	Std. Error	Beta			Tolerance	VIF
1	(Constant)	-.367	.103		-3.560	.000		
	age	.155	.062	.159	2.500	.013	.983	1.017
	Nstage	.146	.052	.214	2.815	.006	.687	1.455
	VascularInvasion	.185	.070	.186	2.656	.009	.815	1.227
	TumorDeposits	.210	.081	.190	2.589	.011	.738	1.354
	PericolicFatInfiltration	.122	.032	.262	3.845	.000	.859	1.164

a. Dependent Variable: LiverMetastasis

Collinearity Diagnostics[a]

Model	Dimension	Eigenvalue	Condition Index	Variance Proportions					
				(Constant)	age	Nstage	Vascular Invasion	Tumor Deposits	PericolicFat Infiltration
1	1	4.216	1.000	.00	.02	.01	.02	.01	.01
	2	.695	2.463	.00	.37	.00	.15	.23	.00
	3	.568	2.725	.00	.09	.00	.27	.55	.01
	4	.362	3.414	.03	.51	.02	.51	.00	.04
	5	.105	6.336	.00	.01	.57	.00	.06	.53
	6	.054	8.811	.95	.02	.41	.06	.14	.42

a. Dependent Variable: LiverMetastasis

Supplementary Figure S4. Collinearity detection.
